# Impact of Coping Veneering Techniques on the Survival of Implant-Supported Zirconia-Based-Crowns Cemented to Hybrid-Abutments: An-In-Vitro Study

**DOI:** 10.3390/bioengineering7040117

**Published:** 2020-09-25

**Authors:** Shareen Hayel Elshiyab, Noor Nawafleh, Usman Khan, Andreas Öchsner, Roy George

**Affiliations:** 1 School of Dentistry and Oral Health, Griffith University, Gold Coast, Queensland 4214, Australia; shelshiyab@just.edu.au; 2 Faculty of Applied Medical Sciences, Jordan University of Science and Technology, Irbid 21110, Jordan; n.nawafleh@just.edu.au; 3 Queensland Health, Brisbane, Queensland 4000, Australia; Sam.dental.prac@gmail.com; 4 Faculty of Mechanical Engineering, Esslingen University of Applied Sciences, 73728 Esslingen, Germany; andreas.oechsner@hs-esslingen.de; 5 School of Dentistry and Oral Health, Griffith University, Gold Coast, Queensland 4000, Australia

**Keywords:** hybrid, implant-supported, fatigue, thermocycling, zirconia-based

## Abstract

The objective of this study is to investigate the influence of veneering technique (hand-layering vs. milling) on the fracture resistance of bi-layer implant-supported zirconia-based hybrid-abutment crowns. Mandibular molar copings were anatomically designed and milled. Copings were then veneered by hand-layering (HL) (n = 20) and milling using the Cad-On technique (LD) (n = 20). Crowns were cemented to zirconia hybrid-abutments. Ten samples of each group acted as a control while the remaining ten samples were subjected to fatigue in a chewing simulator. Crowns were loaded between 50 and 100 N for 1.2 million cycles under simultaneous temperature fluctuation between 5 and 55 °C. Crowns were then subjected to static load a to fracture test. Data were statistically analysed using the one-way ANOVA. Randomly selected crowns from each group were observed under scanning electron microscopy to view fractured surfaces. Study results indicate that during fatigue, LD crowns had a 100% survival rate; while HL crowns had a 50% failure rate. Fracture resistance of LD crowns was statistically significantly higher than that of HL crowns at the baseline and after fatigue (*p* ≤ 0.05). However, fatigue did not cause a statistically significant reduction in fracture resistance in both LD and HL groups (*p* > 0.05). Copings fractured in the LD crowns only and the fracture path was different in both LD and HL groups. According to the results, it was concluded that milled veneer implant-supported hybrid-abutment crowns exhibit significantly higher fracture resistance, and better withstand clinical masticatory loads in the posterior region compared to the hand-layered technique. Also, fatigue application and artificial aging caused no significant strength reduction in both techniques. Clinical significance: Different veneering techniques and materials (hand-layering or milling) act differently to clinical forces and environment and may be prone to early chipping during service. Therefore, practitioners are urged to consider the appropriate veneering protocol for posterior implant-supported hybrid-abutment restorations.

## 1. Introduction

Being the toughest of all dental ceramics [1], zirconium dioxide has been in clinical use for over 10 years. It is reported to be an acceptable coping material for tooth-supported and implant-supported restorations [2,3], and clinical studies have reported a high success rate as an abutment material [4,5,6]. However, reported technical complication in its bi-layer structure include chipping of the veneering ceramics and fracture of the coping [2,5,6,7,8,9,10,11,12,13,14]. This indicates that the veneering ceramic-zirconia interface is the weakest bond. Studies investigating the bond strength between zirconia coping and the veneering ceramics reported mainly cohesive failure within the veneering ceramic [15,16].

Copings of bi-layer zirconia crowns can be veneered using multiple techniques such as conventional hand-layering technique, pressing technique or milling technique using the computer aided design/computer aided manufacturing (CAD/CAM) technology. In the conventional hand-layering technique, a build-up of dentin and enamel porcelain is done directly on the coping. However, in the pressing technique, the veneer is pressed on the coping using an ingot and a special press furnace; this provides superior strength [17] and anatomical characteristics, yet this technique is less aesthetically favourable compared to the latter [18]. Veneering of the zirconia coping can also be done by milling lithium disilicate structure using CAD/CAM, where the coping and the veneering structure are designed using the split-file technology and then milled separately. Following, the milled coping and the veneer structure are fused together using glass-ceramic [19,20].

Satisfactory bonding between the coping and the veneer material can enhance fracture strength and is the key to bi-layered restoration success [8,17]. It is suggested that bond strength of the veneering porcelain to zirconia coping depends on the strength of the porcelain itself [21] and the coefficient of thermal expansion between the veneering material and the coping [22,23]. Aboushelib suggested that the bond between the coping and the veneering ceramic should have a certain minimal strength to prevent chipping of the veneering ceramic under masticatory loading [18].

Being more aesthetically pleasing [24,25], highly biocompatible and less susceptible to plaque accumulation compared to titanium structures [26,27,28], zirconia can also be used as an abutment material for implant-supported restorations in a commercially available standard form or customised form; fabricated by the dental technician using the CAD/CAM technology.

A new approach to achieve aesthetics and strength in implant dentistry is the hybrid-abutment crown approach [29,30]. It is considered relatively new and consists of the following components: (1) hybrid-abutment (an all-ceramic abutment and a Ti-Base), (2) all-ceramic crown. Clinically, the approach was considered to enhance aesthetics and was also regarded as a reliable option in the anterior region [29,30]. Silva et al. [31] suggest that performance of all-ceramic crowns on hybrid-abutments made with lithium disilicate abutments should be adequate clinically. It is also reported that the fracture resistance of a hybrid-abutment all-ceramic crown is influenced by the crown’s structure (monolithic vs. bi-layered) [32], crown’s material [32] and the abutment’s material, manufacturer and design [33]. Elshiyab et al. [34,35] studied the influence of ageing on implant-supported crowns fabricated from zirconia and lithium disilicate and cemented to hybrid abutments. They reported a good performance of this implant-abutment design with no fracture for any of its components. Selz and Vuck [36] considered combining monolithic lithium disilicate crown and a Ti-base (hybrid crowns) a reliable approach in anterior and posterior regions for implant-supported restorations. Also, Edelhoff and Schweiger [37] concluded that higher fracture risk is related to implant connection platform, showing higher fracture risk when having internal connection compared to external connections. Authors suggested that such risk can be avoided when using Ti-bases in the form of a hybrid abutment crown, or a hybrid abutment with a separate crown [37]. However, there is currently insufficient scientific evidence and clinical reports on the applicability of the hybrid-abutment concept in particular and implant-supported single crowns in general [34,35,38].

To date, no information is available about the influence of the veneering technique used in the bi-layer structures on the fracture resistance of such hybrid-abutment crowns. Therefore, this study aimed to evaluate the fracture resistance and post fatigue fracture load of zirconia copings veneered with CAD/CAM milled lithium disilicate structures compared to hand-layered veneered copings when cemented to hybrid-abutments and supported by implants. We hypothesised that implant-supported hybrid-abutment crowns veneered with milled lithium disilicate ceramic would exhibit similar survivability as well as fracture loads to failure compared to hybrid-abutment crowns veneered by hand-layered nano-fluorapatite ceramic. We also hypothesised that fatigue testing in a simulated oral environment would not significantly affect fracture loads for crowns made in either technique.

## 2. Materials and Methods

### 2.1. Sample Preparation

**Figure 1:**
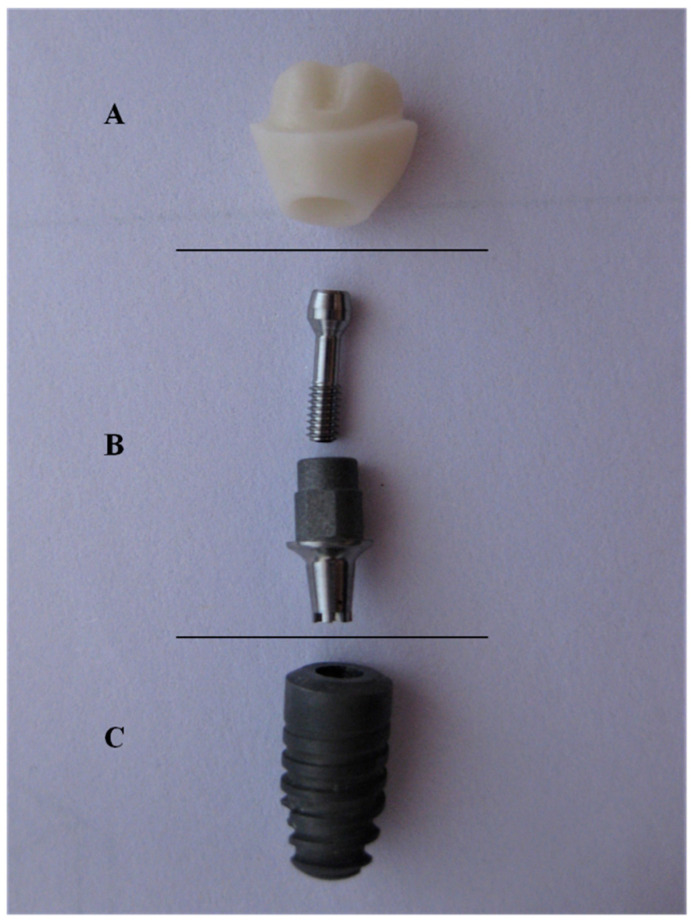
Hybrid-abutment used for this study; (**A**) zirconia abutment (**B**) Ti-Base (with screw) (**C**) Ankylos^®^ implant.

For the purpose of this study, hybrid-abutments (Figure 1) were used and anatomically correct bi-layer crowns for a lower right first molar were veneered to the abutment using two different techniques; the milling technique (IPS e.max^®^ CAD-On) (n = 20) by joining a milled lithium disilicate veneering structure (LD) to the zirconia coping, and hand-layering technique (n = 20) by building up dentin and enamel ceramic on the zirconia (HL). Materials and components used in this study are listed in Table 1.

CAD-On crowns were designed using the split-file technique (3Shape, Copenhagen, Denmark); anatomical zirconia coping was designed first and followed by designing the lithium disilicate veneering structure. The zirconia abutments and the CAD-On design files were then transferred to a 5-axis milling machine (ZENOTEC^®^ select, Wieland Dental, Lindenstraße, Germany) to mill zirconia abutments (n = 40) and zirconia copings (n = 40). Zirconia structures were then sintered in the recommended Programat S1^®^ furnace (Ivoclar Vivadent, Schaan, Liechtenstein) and left to slowly cool. 

#### 2.1.1. Manufacturing Process for CAD-On Crowns (LD)

In a wet milling machine (ZENOTEC^®^ select hybrid, Wieland Dental, Lindenstraße, Germany), LD veneering structures (n = 20) were milled from the same CAD-On design file previously used to mill the zirconia abutments and copings. Afterwards, fitting was checked for: (a) zirconia abutments to the Ti-Base, (b) zirconia copings to zirconia abutments and (c) LD veneering structures on the zirconia copings. Afterwards, IPS e.max^®^ Crystall./Connect capsule was mixed using the Ivomix (Ivoclar Vivadent, Schaan, Liechtenstein) and evenly distributed on the occlusal aspect of the zirconia copings as well as on the fitting surface of the LD veneering structures. Both copings and veneering structures were then joined together using Crystall./Connect and any excess material was removed from the circular fusion joint. To verify the correct join between the coping and the veneering structure, all LD crowns were checked for occlusion in an articulator. The recommended Fusion/Crystallisation firing of the LD crowns was conducted in a Programat EP 3010^®^ furnace (Ivoclar Vivadent, Schaan, Liechtenstein). Finally, LD crowns were glazed and recommended glazing firing was also conducted in the same Programat 3010^®^ furnace.

#### 2.1.2. Manufacturing Process for Hand-Layered Crowns (HL)

Using IPS e.max^®^ Ceram (Ivoclar Vivadent, Schaan, Liechtenstein), hand layering of the zirconia copings was done by a dental technician with 20 years of experience. The silicone index of an LD crown was taken and used during the porcelain build-up process of dentin and enamel porcelain of the HL crowns; to ensure standardisation of both anatomical shape and thickness of the layering porcelain in both LD and HL crowns. Then, all crowns were glazed as per the manufacturer recommendation using the IPS e.max Ceram glaze. Firing of the dentin porcelain, enamel porcelain and glazing was conducted as per manufacturer recommendation in a Programat 3010^®^ furnace (Ivoclar Vivadent, Schaan, Liechtenstein).

### 2.2. In-Vitro Testing

To cement the crowns to the Ti-Base, the Ti-Base access hole was first filled with a temporary restorative material (Fermit N; Ivoclar Vivadent AG, Schaan, Liechtenstein), and zirconia abutments were then luted to the Ti-Base using a self-curing dental luting composite (Multilink^®^ Hybrid Abutment, Ivoclar Vivadent, Schaan, Liechtenstein) as per the manufacturer instructions.

Twenty-four hours later, both LD and HL crowns were adhesively luted to the zirconia abutments using Multilink^®^ Automix (Ivoclar Vivadent, Schaan, Liechtenstein) as per manufacturer instructions. Specimens were stored in distilled water at 37 °C for a minimum of 7 days prior to testing [39].

Both LD (n = 20) and HL (n = 20) groups were then divided into two subgroups (n = 10) according to the testing to be conducted; (1) control group (non-fatigued) crowns to be subjected to static load to fracture (SLF) in a universal testing machine, (2) test group (fatigued) to undergo thermal cycling mechanical loading in a chewing simulator prior to the SLF test.

**Figure 2:**
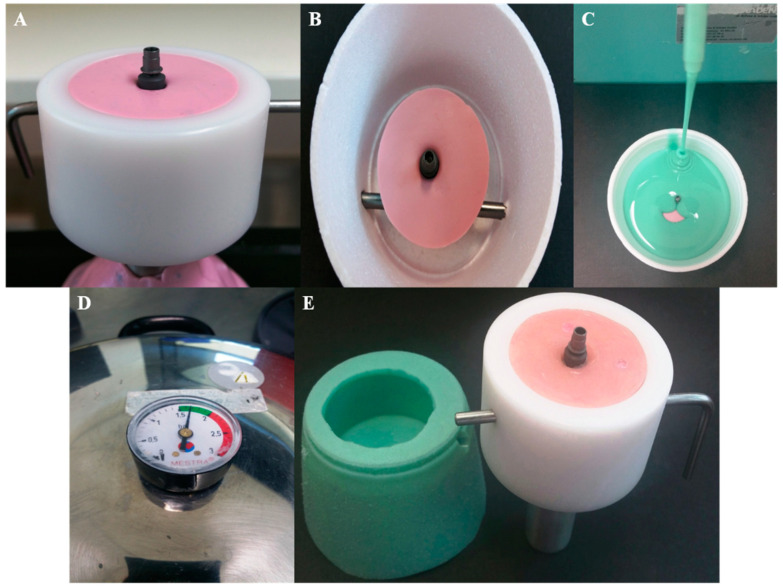
Fabrication of the sample cup holder for fatigue testing; (**A**) implants were screwed into heavy putty (Coltene whaledent, Altstatten/Switzerland). (**B**) Ti-Base abutment torqued to implant and ready to pour duplicate material. (**C**) Silicone duplicate material (Exaktosil N21, Bredent) poured into the cup. (**D**) Silicone replica put to set in a pressure pot to avoid porosity. (**E**) Silicone replica of the sample cup with the implant and Ti-Base abutment inverted and acrylic resin (Palapress vario, Heraeus Kulzer Wehrheim, Germany) poured in the mold and checked in the original chewing simulation sample cup for fitting.

To prepare samples for fatigue testing (Figure 2) in the chewing simulator (CS-4.8; SD Mechatronik GmbH, Feldkirchen-Westerham, Germany), all implants were inserted in acrylic resin base (Palapress vario, Heraeus Kulzer Wehrheim, Germany) up to the first thread to simulate clinical procedures; the acrylic resin modulus of elasticity (12 GPa) approximates that of human bone (18 GPa) [40]. In addition, a jig especially designed for the SLF test to fit samples of study subgroups and to ensure that samples are stable during compressive loading as well as to prevent any lateral movement of samples; samples had no angulations and the load was applied vertically using a 6-mm diameter stainless steel spherical indenter.

#### 2.2.1. Thermal Cycling Mechanical Loading (Fatigue)

**Figure 3:**
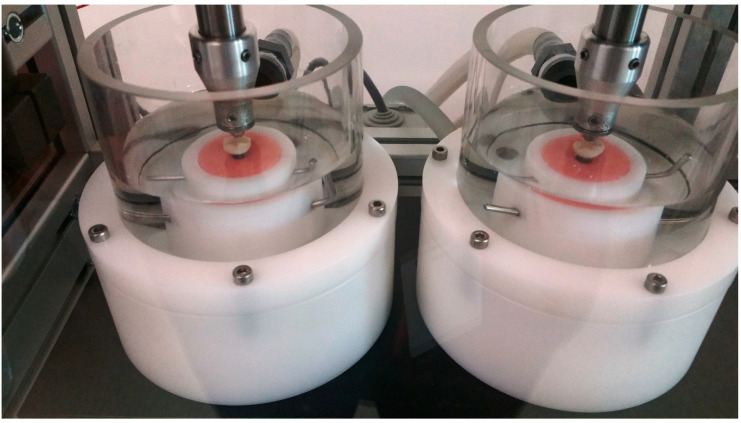
Crowns undergoing thermal cycling in a chewing simulator during fatigue testing.

Using a 6-mm diameter stainless steel spherical indenter and a loading frequency of 1.2 Hz, crowns were loaded for 1,200,000 cycles in a chewing simulator to simulate 5 years of clinical service [40,41,42]. The loading protocol was as follows: crowns initially loaded with 50 N for 250,000 cycles, the following 500,000 cycles were loaded with 100 N, and the last 450,000 cycles were loaded with 50 N. To simulate aspects of natural masticatory settings during testing [43], indenters were positioned with a mouth opening of 6 mm to simulate natural masticatory function, and 0.5 mm lingual to the disto-buccal cusp tip and sliding 0.3 mm lingual [44] to the central fossa. Throughout the testing (Figure 3), crowns underwent simultaneous thermal cycling between 5 and 55 °C in distilled water (5118 thermal cycles, 60 s dwell time, and 15 s pause time to empty the chambers). At the end of each loading stage, each crown was inspected under an 8× magnification using endodontic microscopy (GLOBAL A-Series™, Global Corp, MO, USA) for the presence of any chipping, cracks or fractures. All crowns that had visual evidence of chipping or cracking were removed from further fatigue testing.

#### 2.2.2. Compressive Static Load to Fracture Testing (SLF)

**Figure 4:**
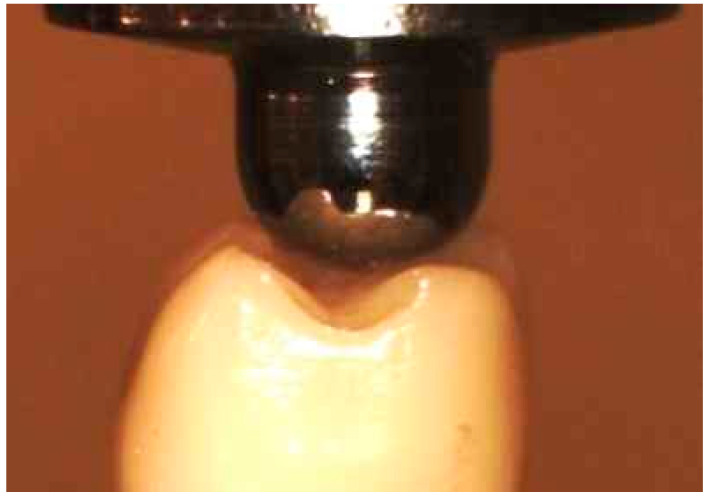
Position of the indenter during the compressive testing.

Both control groups (not subjected to Thermal Cycling Mechanical Loading (TCML) were subjected to compressive SLF loading test (Instron, Model 3367, Norwood, MA, USA) until failure. The compressive loading was applied on the crowns at three points (the triangular ridges of both lingual cusps and the disto-buccal cusp, as shown in Figure 4, at a crosshead speed of 1 mm/min [45].

### 2.3. Scanning Electron Microscopy (SEM)

After fracture resistance testing, representative crowns from each group were observed by using the TESCAN scanning electron microscopy (SEM) (Mira 3XMU, Kohoutovice, Czech Republic), to check the fractured surface condition and highlight any differences between the groups (if present). Sputter coating of the crown was done with gold (Leica EM SCD050, Wetzlar, Germany) to a thickness of approximately 10 µm prior to imaging.

### 2.4. Statistical Analysis

Statistical analysis of the data was conducted using the SPSS software (version 24.0; IBM, Chicago, IL, USA). Skewness and Kurtosis tests and P-P plots were used to check the normality of data distribution. One-Way Analyses of Variance (ANOVA) was used to compare means and to evaluate statistical significance between all study groups. A post-hoc assessment was performed using the Tukey HSD test. T-test was also conducted to analyse data of the HL crowns of the fatigued group (survived vs. failed). A *p* value equal to or less than 0.05 was set to indicate statistical significance.

## 3. Results

### 3.1. Thermal Cycling Mechanical Loading (Fatigue)

**Figure 5:**
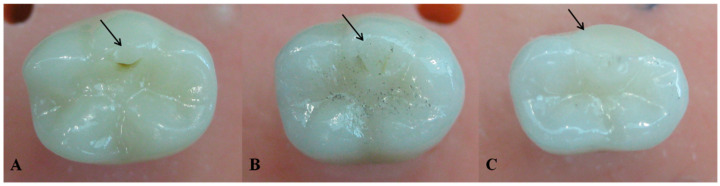
Wear facets at the indenter occlusal contact upon completion of the chewing simulation (arrows). (**A**) Milled lithium disilicate veneer, (**B**) hand-layered veneer, and (**C**) arrow indicating the chipping on the disto-buccal cusp at the indenter occlusal contact during chewing simulation.

No implant fractures occurred during fatigue in the chewing simulator in both LD and HL groups. Also, no failure occurred for the LD crowns during chewing simulation. Nevertheless, HL veneered crowns failed by means of chipping under the indenter contact point during chewing simulation and at a different number of cycles; two crowns failed during the first stage of the loading protocol under 50 N; at the end of 250,000 cycles. Also, three crowns failed during the second stage of the loading protocol less than 100 N; one failed at 418,000 cycles and the other two failed at the end of the 500,000 cycles. Wear facets on the occlusal contact of the indenter were evident in both LD and the survived HL crowns (Figure 5).

### 3.2. Static Load to Fracture (SLF)

The ultimate fracture load values (standard deviation) recorded in newtons for un-fatigued crowns were as follows:

Crowns veneered with milled lithium disilicate (n = 10):

F = 4625 N (+/−507)(1)

Hand-layered veneered crowns (n = 10):

F = 1640 N (+/−130)(2)

On the other hand, the ultimate fracture loads values (standard deviation) recorded in newtons fatigued crowns were as follows:

Crowns veneered with milled lithium disilicate (n = 10):

F = 3897 N (+/−446)(3)

Hand-layered veneered crowns (n = 10):

F = 1258 N (+/−66)(4)

**Figure 6:**
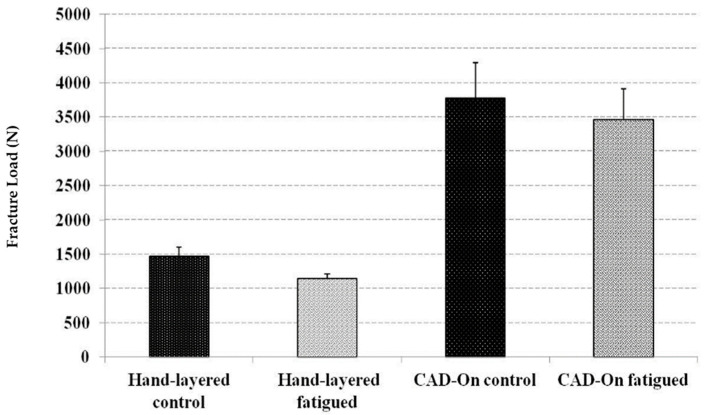
Mean and standard deviation of fracture loads in Newtons (N) for all study groups.

Mean and standard deviation of fracture loads in Newtons (N) for all study groups are presented in Figure 6. Normal distribution of data was confirmed by Skewness and Kurtosis as well as the P-P Plots. In addition, one-way ANOVA analysis showed that LD crowns had statistically significant higher fracture resistance (*p* ≤ 0.05) than HL crowns. However, there was no statistically significant difference between the control and the fatigued crowns in both groups.

Upon conducting the SLF test, it was observed that crowns of HL subgroups had a cohesive fracture of the veneering on the lingual cusps, which extended to the coping-ceramic interface with no fracture of the coping. On the contrary, both veneer structure and copings fractured in LD subgroups. In addition, no ceramic abutments, Ti-Base or implants were fractured and no screw loosening occurred in any of the groups.

### 3.3. Scanning Electron Microscopy (SEM)

**Figure 7:**
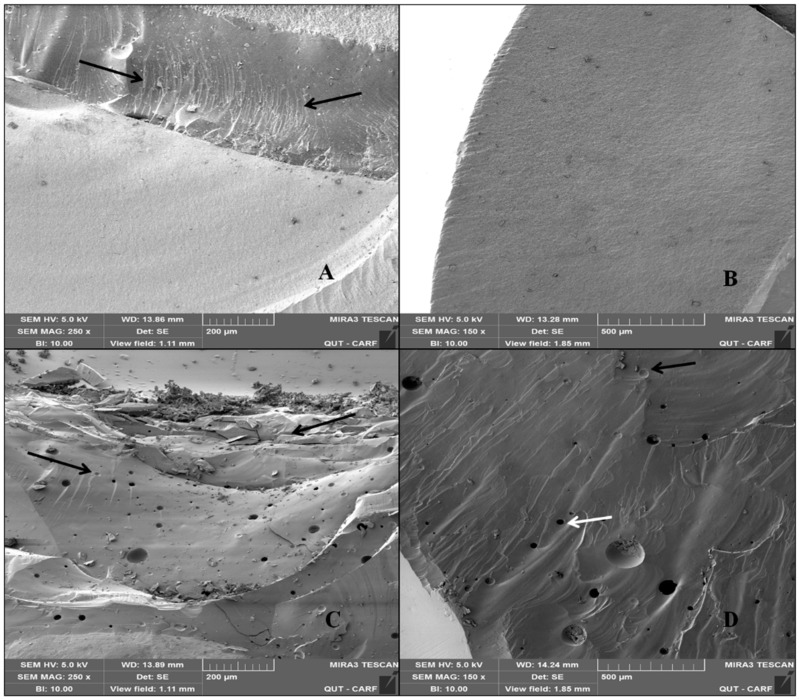
Representative SEM images of fractured surfaces for (**A**) milled lithium disilicate veneer at 250× showing hackle lines (arrows) and (**B**) buccal view of lithium disilicate veneer 150×, (**C**) view of the occlusal surface shows catastrophic chipping of the veneering porcelain (pointer) in the hand-layered veneer at 250×, (**D**) a buccal view of the hand-layered veneer showing wake hackles as a distinctive indicator of crack propagation (black arrow) as well as pores (white arrow).

Representative SEM images of fractured surfaces are presented in Figure 7. Presence of pores in the veneering layer was evident. Hackles and wake hackles were also present on both LD and HL fractured surfaces, which indicates the orientation of the crack.

## 4. Discussion

This study looked at bi-layer zirconia-based implant-supported hybrid-abutment crowns in the posterior region. Our results showed that zirconia-based copings for hybrid-abutment crowns veneered with a lithium disilicate veneer had significantly higher fracture load to failure compared to the hand-layered veneer. Also, fatigue did not cause significant reduction in fracture resistance for crowns veneered in either technique with milled lithium disilicate veneers resulting in 100% survival rate. Nonetheless, some hand-layered veneered crowns failed during fatigue testing by means of chipping. These findings reject our hypothesis that hybrid-abutment crowns made of zirconia copings and veneered with milled lithium disilicate exhibit similar fracture loads and survivability compared to crowns with hand-layered veneers. However, the hypothesis that fatigue testing will have no significant effect on the fracture load of hybrid-abutment crowns veneered in both techniques was accepted. Past experimental or clinical studies provide no semblance to the current work. Thus, results of this study may be hard to directly compare to other studies conducted on hybrid-abutment crowns.

Fracture resistance of crowns with CAD/CAM lithium disilicate veneer was significantly higher than that of the hand-layered veneers. Studies on tooth-supported crowns reported the same results with CAD/CAM veneered zirconia coping, displaying significantly higher fracture loads compared to the hand-layered veneers [46,47,48]. Fracture load values of CAD/CAM lithium disilicate veneers reported in all the previous studies and in the current studies are all higher than the maximum chewing forces [49,50,51].

Five crowns of the hand-layer veneered group suffered chipping of the veneer during fatigue testing on the cusp where the indenter was loaded. The high veneer chipping rate of hand-layered zirconia-based restorations was also reported in a previous study which compared both veneering methods in tooth-supported crowns [20]. Kassem et al. [52] reported that cyclic loading caused cracks in the zirconia based crowns. Further testing reported micro-leakage to the dentine; this was caused by the cracks present in the zirconia-based crowns after cyclic loading [52]. Although the study was done on natural teeth, this micro-leakage might also occur in chipped or cracked implant-supported crowns, which can possibly extend to the abutment and the implant leading to peri-implantitis.

Such significant difference in fracture load values between both veneering techniques might be due to differences in both veneering materials and fabrication techniques used; while a lithium disilicate veneer has a flexural strength of 360 MPa, the veneering ceramic used in the hand-layering technique has a flexural strength of 90 MPa making the later more prone to failure at low loads during mastication [53]. Also, lithium disilicate veneers were milled using the CAD/CAM technology, which is a controlled technique following certain procedure where human error is not as a significant factor [54]; that allows for very minor flaws and structural defects in the veneering ceramic and results in an enhanced strength. In addition, the zirconia coping is subjected to a few firing cycles, which reduces the likelihood of thermal fatigue [54]. However, the conventional hand-layering technique is operator-dependant and requires superior technical expertise to achieve aesthetics and despite following the ultimate attention to applying the layering details and firing procedures; porosity, voids and maybe micro gaps at the coping-ceramic interface is most likely to occur during layering [55,56] and may be responsible for veneer fracture.

In the oral cavity, chipping usually occurs after exposure to a localised load on one or more cusp during mastication while undergoing aging at the same time [47]. For the purpose of replicating real word clinical scenarios, anatomically correct crowns with coping and veneering structure of all the groups were fabricated according to the minimum recommended thickness used for clinical restorations. Also, identical veneering thickness and occlusal morphology was done for all tested crowns. In addition, laboratory procedures for milling the copings and veneering them in the previously mentioned techniques, as well as the cementation were done as per manufacturer recommendations. Moreover, crowns were exposed to a testing method that closely mimics the clinical scenario, which was achieved by using the chewing simulator to apply mouth-motion fatigue loads as well as applying simultaneous temperature fluctuation. The mouth-motion fatigue causes failures, which start from the indenter loading point in the outer and inner cone crack forms and then extends to the veneer/core interface [57], which closely simulates the failure behaviour of crowns in the clinical condition [58]. Although not statistically significant, crowns, which underwent aging by means of thermocycling, had lower mean fracture load values. The wear facets that were observed on the surface of all crowns where the indenter loading occurred may have caused inner cracks, weakening the veneering material and reducing the fracture resistance; this observation was also reported by Schmitter et al. [47].

Agreeing with previous studies [34,35], neither implants nor any of the hybrid-abutment components fractured/failed in this study. Loads applied to the implant-supported crown can be transmitted to other parts of the crown system, which would increase the stresses on both the ceramic abutment and abutment-implant junction. The current study findings suggest that adhesive bonding of the zirconia abutment to the Ti-Base can withstand occlusal forces and, therefore, would be considered a favourable clinical approach.

## 5. Conclusions

Within the limitation of the current study, it was concluded that veneering hybrid-abutment zirconia-based crowns using a milled lithium disilicate structure creates a crown with high fracture resistance properties, and may result in highly clinically reliable restorations. Also, we concluded that crowns veneered using the conventional hand-layering technique are likely to fail by chipping of the veneering layer early during clinical service, which makes them unfavourable and less cost effective for both the clinician and patient. Furthermore, it was concluded that the combination of zirconia abutment to a Ti-Base (hybrid-abutment) has an adhesive bond that is unlikely to fail during clinical service.

Results of this study are only an indication of the hybrid implant abutment system’s behaviour in an in-vitro environment; we suggest that short and long-term clinical trials are necessary to assess implant-supported hybrid-abutment crowns veneered in both veneering techniques.

## Figures and Tables

**Table 1:** Study components and materials.

Component	Description	Manufacturer
Forty implants	5.5 mm diameter Ankylos^®^ C/X titanium implants	DENTSPLY–Friadent GmbH, Mannheim, Germany
**Forty Hybrid-Abutments**		
1. Titanium base (Ti-Base) 2. Zirconia abutments	1. Internal Ankylos^®^ compatible Ti-Base; 1.00 mm hex screw, 4 mm height and 0° angulations 2. Zirconia abutments with 1.0 mm depth shoulder	1. Dess, Dental Smart Solutions, Montcada, Spain 2. Zenostar, Ivoclar Vivadent, Lichtenstein, Germany
Forty copings	Anatomically designed as per manufacturer recommendations (0.5 mm circular and 0.7 occlusal) and milled from pre-sintered zirconia discs	Zenostar, Ivoclar Vivadent, Lichtenstein, Germany
Veneering material	1. Hand-layered nano-fluorapatite ceramic. IPS e.max Ceram (0.7 mm circular and 0.7 occlusal) 2. Milled lithium disilicate blocks, IPS e.max CAD (0.7 mm circular and 0.7 occlusal)	Ivoclar Vivadent, Lichtenstein, Germany
